# Transient Cold Agglutinins in a Patient With COVID-19

**DOI:** 10.7759/cureus.12751

**Published:** 2021-01-17

**Authors:** Jasmeet Kaur, Swathi Mogulla, Rafiullah Khan, Geetha Krishnamoorthy, Sandeep Garg

**Affiliations:** 1 Internal Medicine, St. Joseph Mercy Oakland Hospital, Pontiac, USA; 2 Hematology and Oncology, University of Cincinnati Medical Center, Cincinnati, USA; 3 Hematology and Medical Oncology, St. Joseph Mercy Oakland Hospital, Pontiac, USA

**Keywords:** covid -19, severe acute respiratory syndrome coronavirus 2, cold agglutinins, blood coagulation, anemia

## Abstract

Coronavirus disease 2019 (COVID-19) infection has been associated with various complications such as acute respiratory distress syndrome, acute kidney failure, myocardial infection, and thromboembolism. Cold agglutinin syndrome (CAS) has been associated with other viral infections such as Epstein-Barr virus (EBV), but there have been only a few reports of cold agglutination associated with COVID-19. In this report, we describe a case of transient cold agglutinin elevation in a COVID-19-infected patient. A 61-year-old man with hypertension, diabetes mellitus, and end-stage renal disease (ESRD) presented with shortness of breath, cough, and lethargy for five days. A clinical diagnosis of COVID-19 infection was made. The COVID-19 RNA qualitative real-time polymerase-chain-reaction (PCR) assay tested positive. During the hospital stay, he had progressive dyspnea requiring intubation and mechanical ventilation. During the third week of hospital stay, an acute drop in the hemoglobin (Hb) level to 4.5 g/dl (baseline Hb: 9 g/dl) was observed. The workup for acute anemia revealed a positive result for cold agglutinins, direct antibody test (C3d), and agglutination of the red blood cells were apparent on the peripheral blood smear. Further, cold agglutinin titers peaked during the third week of the onset of illness and significantly declined during the fifth week. These observational findings indicate that cold agglutinin titers might correlate with the disease activity.

## Introduction

Cold agglutination is a type of extravascular autoimmune hemolytic anemia (AIHA). This condition is characterized by the presence of autoantibodies, also known as cold agglutinins, which causes agglutination at a temperature as low as 3-4 °C when the red blood cells circulate in the cooler parts of the body [1]. Primary cold agglutinin disease (CAD) may occur in the absence of an underlying condition. In contrast, secondary cold agglutinin syndrome (CAS) is associated with infections, autoimmune conditions, lymphoproliferative diseases, and Waldenstrom macroglobulinemia [2]. Mycoplasma infection and Epstein-Barr virus (EBV) infection are the most common causes observed [3]. Case reports have described cold agglutinins in the setting of other infections such as HIV, rubella virus, influenza viruses, or varicella-zoster virus (chickenpox) as well [3]. Not all individuals with these infections who develop cold agglutinins will have clinically significant hemolysis. We describe a case of CAS observed in an adult male patient with severe acute respiratory syndrome coronavirus 2 (SARS-CoV-2) infection; he presented with symptoms of sepsis and hypoxemia to the emergency department and developed cold agglutinins and acute anemia.

## Case presentation

A 61-year-old man with hypertension, type 2 diabetes mellitus, hypercholesterolemia, end-stage renal disease (ESRD), hemodialysis-dependent, anemia of chronic disease, coronary artery disease, paroxysmal atrial fibrillation, and obesity presented with complaints of dry cough, fever, lethargy, and worsening shortness of breath for five days. He was found to be in mild respiratory distress. The patient was febrile at 38 °C, had a blood pressure of 156/86 mmHg, heart rate of 74 beats per minute, a respiratory rate of 22 breaths per minute, and was saturating 89% on room air. On physical examination, there was no hepatomegaly and lymphadenopathy, but there were reduced breath sounds at lung bases with bibasilar crackles. Laboratory evaluation revealed a white blood cell count of 5,700 cells/µL (normal range: 3,700-1,100 per µL); hemoglobin (Hb) of 9.0 g/dL (normal range: 13-18 g/dL); and troponin I of 0.04 ng/mL (other lab values are summarized in Table 1). A chest radiograph showed significant bilateral lung infiltrates with pulmonary vascular congestion. Nasopharyngeal swab test detected SARS-CoV-2, a qualitative real-time polymerase chain reaction (PCR) assay. The following day, the patient developed progressive hypoxic respiratory failure and required intubation and mechanical ventilation. He was given oral hydroxychloroquine 400 mg on the first day followed by 200 mg twice daily and azithromycin 500 mg once daily for five days. He also received methylprednisolone 60 mg intravenously (IV) daily for seven days. He was managed with anticoagulation therapy for atrial fibrillation with a rapid ventricular rate. He continued to receive supportive care, including hemodialysis and mechanical ventilation.

**Table 1:** Laboratory data RBC: red blood cells; MCV: mean corpuscular volume; ALP: alkaline phosphatase; ALT: alanine aminotransferase; AST: aspartate aminotransferase; LDH: lactate dehydrogenase

Variable	Reference range (adult)	Day 1	Day 6	Day 8	Week 3	Week 5	Week 7
Hemoglobin (g/dl)	13.0-18.0	9	5.6	4.5	7.5	7.9	9.2
Hematocrit (%)	39.0-50.0	27.8	18.4	23.5	23.6	27.3	29.7
White cell count (10^3^/µl)	3.7-11.0	5.7	17.7	24.9	34.9	8.6	8.2
Differential cell count (10^3^/l)							
Neutrophils	1.5-10	4.4	16.1	21.4	33.9	7.4	5.9
Lymphocytes	1.0-3.5	0.6	0.9	2.2	0.7	0.7	1.1
Eosinophils	0.0-0.5	0.0	0.0	0.2	0.0	0.0	0.1
Basophils	0.0-0.2	0.0	0.0	0.0	0.0	0.0	0.0
Monocytes	0.0-1.0	0.7	0.6	1.0	0.3	0.5	1.0
Platelet count (10^3^/µl)	140-440	183	250	233	199	157	293
RBC (10^6^/µl)	4.00-6.00	3.07	1.9	1.58	2.43	2.80	3.09
MCV (fl)	80-99	91	91	97	92	97	96
ALP (U/liter)	38-126	90	94	126	140	99	102
Bilirubin (mg/dl)							
Total	0.0-1.6	1.3	1.8	3.1	4.1	0.9	0.8
Direct		-	-	0.6	-	-	-
ALT (U/liter)	10-63	21	24	25	27	20	17
AST (U/liter)	15-41	35	36	46	19	14	14
Albumin (g/dl)	3.5-4.8	2.6	1.7	1.7	2	2.2	2.6
Total protein (g/dl)	6.1-7.9	6.5	5.8	5.5	5.6	56	6.9
Globulin (g/dl)	2.3-3.5	39	4.1	3.8	3.6	3.4	4.3
C-reactive protein (mg/liter)	<0.5	20.6	26.7	18.0	14.0	13.2	2.9
LDH (U/L)	98-192	276	486	500	504	199	147
Ferritin (ng/ml)	24-336.0	959	2,257	1,560	1,102	892	869

**Figure 1:**
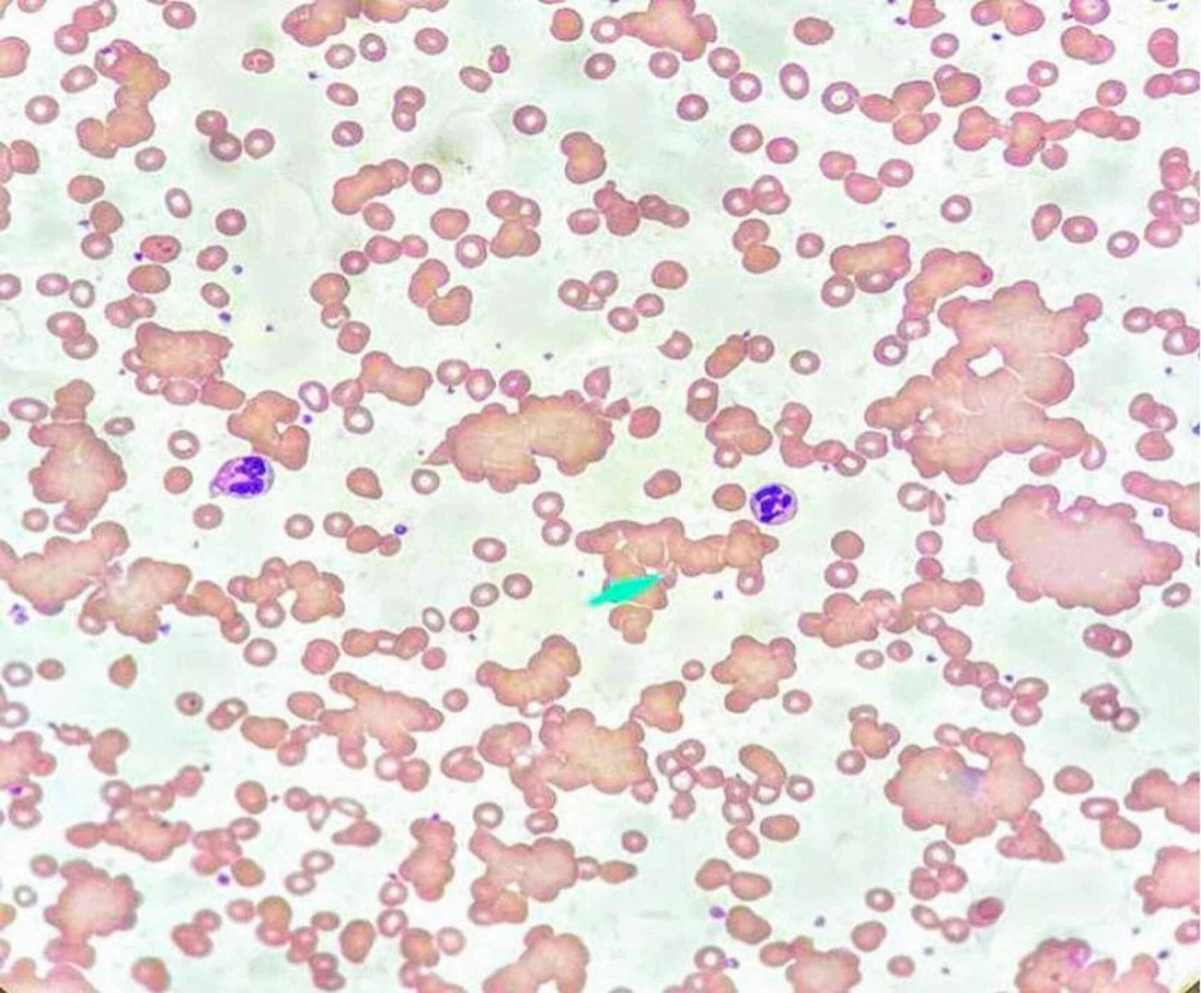
Peripheral blood smear at 7 degree Celsius Image courtesy of American Society of Hematology (ASH) Image Bank; 2020 (08/17) 63419

**Figure 2:**
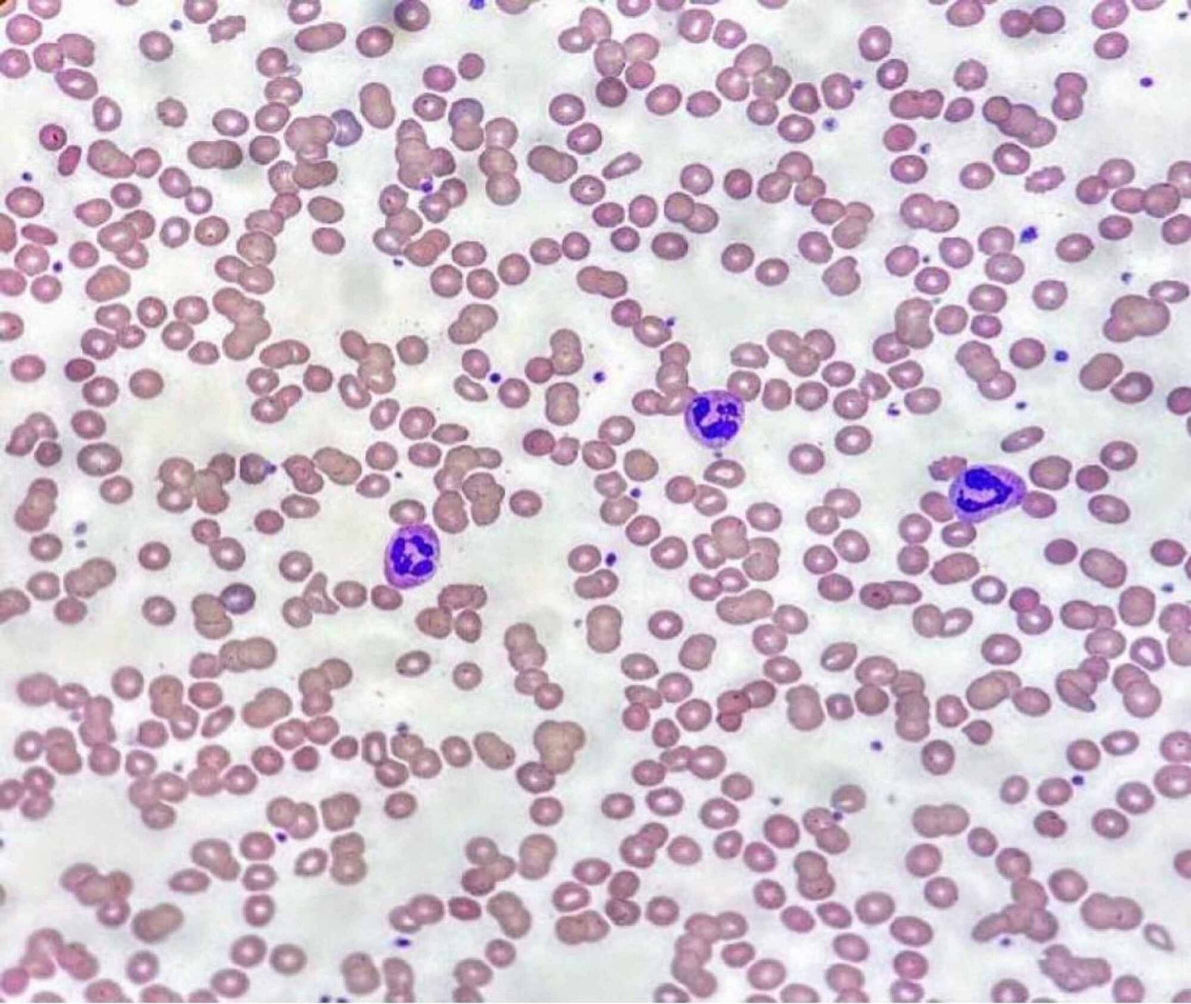
Peripheral blood smear at 37 degree Celsius Image courtesy of American Society of Hematology (ASH) Image Bank; 2020 (08/17) 63150

On day five of hospital stay, an acute drop in Hb to 6.2 g/dL was observed. Hb continued to drop during the following few days, reaching a nadir of 4 g/dL on day eight. There were no signs of active bleeding. The workup for acute anemia revealed serum iron levels of 59 mcg/dL (normal range: 35.5-44.9); ferritin level was found to be 1,250 ng/mL (normal range: 24-336), and iron saturation was 22% (normal range: 20-55%). The absolute reticulocyte counts were 0.1200 M/µL (normal range: 0.01600-0.1000 M/µL), and the reticulocyte index was 1.84% (normal range: 0.5-2.5%). Total bilirubin was 3.1 mg/dL (normal range: 0.0-1.6 mg/dL) and direct bilirubin was 0.6 mg/dL; lactate dehydrogenase (LDH) was 500 U/L, and D-dimer was 3,890 ng/ml. Peripheral blood film showed extensive agglutination of red blood cells (RBCs) at a low temperature of 7 °C (Figure 1), but there was no agglutination on rewarming the blood sample to 37 °C (Figure 2). His direct antibody testing was positive (2+) for anti-complement (C3d) direct antiglobulin. Cold agglutinin titers were elevated at 1:160. Further testing for his hemoglobin free plasma level found it to be elevated: 192 mg/dL (normal range: <8.0 mg/dL), but the repeat value two weeks later was lower (10.5 mg/dL). Urine analysis was positive for 3+ blood, but urine hemosiderin was negative. Retroperitoneal and complete abdominal ultrasound showed no active bleeding or hematoma. A fecal occult blood test was negative. Fecal immunohistochemical testing was not done. Gastrointestinal (GI) workup with upper GI endoscopy and colonoscopy was not performed because of coronavirus disease 2019 (COVID-19) positivity, and there was no evidence of frank bleeding. Subsequently, his Hb level stabilized. The lab workup for other causes of cold agglutinins, such as mycoplasma, EBV, influenza A and B, legionella, and mycoplasma were negative. Respiratory syncytial virus (RSV), hepatitis viral panel, and HIV 1/2 antibody were not detected. The rheumatologic workup was done. Serum protein electrophoresis did not detect monoclonal gammopathy, and abdominal ultrasonography did not reveal hepatosplenomegaly or lymphadenopathy. He received eight units of blood transfusions and three units of cryoprecipitate for acute anemia and hypofibrinogenemia from consumptive coagulopathy, which has been reported in SARS-CoV-2 infection [4,5]. The patient’s fibrinogen levels were noted to be low: 93 mg/dl (normal range: 195-495 mg/dl). Repeat cold agglutinin titer during the fifth week showed a significantly low titer of 1:40. The patient clinically improved, and his Hb level stabilized to 9 g/dL during the fourth and fifth weeks of hospital stay.

## Discussion

This report discussed a case of transient cold agglutinins and acute anemia in a patient diagnosed with SARS-CoV-2 infection. His reticulocyte count could have been low due to his ESRD. CAS has been commonly reported secondary to mycoplasma and infectious mononucleosis with evidence of extravascular hemolysis and anemia [4]. Our literature review showed that low titer cold agglutinin has been reported in infection with trypanosomiasis, atypical pneumonia, and Listeria monocytogenes [6]. Finland et al. and Peterson et al. have reported the presence of cold agglutinins in their study on atypical pneumonia patients [7,8]. Among the 200 patients in that study, 137 patients were found to have cold agglutinins with titers of 1:40 or above. They identified the presence of cold agglutinin titers in the atypical pneumonia patients. Subsequently, a study done by Finland et al. demonstrated a maximum of cold agglutinin titers observed during the middle of the second week and the middle of the fourth week [9]. Titers drop rapidly after reaching the peak value. Significantly lower titers are observed in the third and fifth weeks from the onset of symptoms. The highest titer has been observed to be anywhere from 1:40 to 1:1280. The levels of titers have been found to be correlated with the severity of symptoms, duration, the extent of the pulmonary lesion, and fever spike; 11 out of the total of 200 patients were found to have hemolytic anemia. A similar trend was noted related to mycoplasma infections in other studies [10,11].

After conducting extensive research of published literature in the English language, we herein presented the third report of elevated cold agglutinins in a COVID-19-infected patient, who successfully recovered from life-threatening respiratory failure. A case series of seven patients infected with SARS-CoV-2 reported by Lazarian et al. found that the patients had AIHA [12]. Three patients had cold AIHA and four patients had warm AIHA, which was noticed after the onset of the symptoms of SARS-CoV-2 infection. These patients were managed with blood transfusion and corticosteroids. Hematologic disorders such as immune thrombocytopenia and anti-phospholipid syndrome associated with COVID-19 have been observed in these patients [13,14]. Another case reported by Zagorski et al. demonstrated cold agglutination and hemolytic anemia associated with COVID-19 [15]. The pathophysiology of COVID-19 is not a fully established, cytokine storm with a hypercoagulable state, and an increased incidence of venous thromboembolism has been postulated [16,17]. This hematologic dysfunction with proinflammatory infection conditions, as seen in COVID-19, leads to hemolysis. The present case demonstrated transient cold agglutinins and acute anemia in a patient diagnosed with SARS-CoV-2 Infection. However, no evidence of hemolysis was established in this case. Our patient uniquely presented transient cold agglutinin titers elevation but not cold agglutinin disease. Finland et al. have reported the presence of cold agglutinins in patients with atypical pneumonia; only 11 patients among the 200 patients observed were found to have hemolysis [18]. In the present case, it was observed that the elevated cold agglutinin titers peaked during the second week of onset of illness and significantly decreased during the fifth week. Acute anemia and cold agglutination were observed, but the reticulocyte count was not significantly elevated. Therefore, evidence of hemolysis could not be fully established. In the previously reported cases among COVID-19 patients, two out of three patients had monotypic lymphocyte populations, but this was not observed in our case. Although the acute drop of Hb was not related to CAS in our patient, the incidental finding of elevated cold agglutinins was noted.

## Conclusions

We concluded that CAS or transient cold agglutination in COVID-19 might be correlated with disease severity. The patient’s Hb levels stabilized and cold agglutinin titers were trending down with clinical improvement. We wanted to highlight that the association between CAS and transient cold agglutination with COVID-19 should be seriously considered. The exact pathophysiology of CAS in COVID-19 is not known, but the focus in the management of CAS should be to treat the underlying cause. There is a need for continuous observation of CAS in COVID-19 patients to fully establish the association. The acute drop in Hb levels in COVID-19 patients might be associated with CAS hemolysis, which is otherwise postulated to be consumption coagulopathy. Cold agglutination can be observed in virus infection without significant hemolysis. SARS-CoV-2 infection should be considered as one of the causes behind the development of cold agglutination.
